# Fyn Kinase-Mediated PKCδ Y311 Phosphorylation Induces Dopaminergic Degeneration in Cell Culture and Animal Models: Implications for the Identification of a New Pharmacological Target for Parkinson’s Disease

**DOI:** 10.3389/fphar.2021.631375

**Published:** 2021-04-28

**Authors:** Hariharan Saminathan, Anamitra Ghosh, Danhui Zhang, Chunjuan Song, Huajun Jin, Vellareddy Anantharam, Arthi Kanthasamy, Anumantha G. Kanthasamy

**Affiliations:** Parkinson Disorders Research Program, Iowa Center for Advanced Neurotoxicology, Department of Biomedical Sciences, Iowa State University, Ames, IA, United States

**Keywords:** Fyn, PKCδ, neurodegeneration, phosphorylation, kinase

## Abstract

Oxidative stress, neuroinflammation and apoptosis are some of the key etiological factors responsible for dopamin(DA)ergic degeneration during Parkinson’s disease (PD), yet the downstream molecular mechanisms underlying neurodegeneration are largely unknown. Recently, a genome-wide association study revealed the *FYN* gene to be associated with PD, suggesting that Fyn kinase could be a pharmacological target for PD. In this study, we report that Fyn-mediated PKCδ tyrosine (Y311) phosphorylation is a key event preceding its proteolytic activation in a 1-methyl-4-phenyl-1,2,3,6-tetrahydropyridine (MPTP) model of Parkinsonism. MPP^+^/MPTP induced Fyn kinase activation in N27 DAergic neuronal cells and the mouse substantia nigra. PKCδ-Y311 phosphorylation by activated Fyn initiates the apoptotic caspase-signaling cascade during DAergic degeneration. Pharmacological attenuation of Fyn activity protected DAergic neurons from MPP^+^-induced degeneration in primary mesencephalic neuronal cultures. We further employed Fyn wild-type and Fyn knockout (KO) mice to confirm whether Fyn is a valid pharmacological target of DAergic neurodegeneration. Primary mesencephalic neurons from Fyn KO mice were greatly protected from MPP^+^-induced DAergic cell death, neurite loss and DA reuptake loss. Furthermore, Fyn KO mice were significantly protected from MPTP-induced PKCδ-Y311 phosphorylation, behavioral deficits and nigral DAergic degeneration. This study thus unveils a mechanism by which Fyn regulates PKCδ′s pro-apoptotic function and DAergic degeneration. Pharmacological inhibitors directed at Fyn activation could prove to be a novel therapeutic target in the delay or halting of selective DAergic degeneration during PD.

## Introduction

Parkinson’s disease (PD) is a neurodegenerative disorder characterized by disabling motor deficits such as resting tremor, muscular rigidity, scarce voluntary movements and postural instability (Poewe et al., 2017; Elkouzi et al., 2019). The 1-methyl-4-phenyl-1,2,3,6-tetrahydropyridine (MPTP)-induced Parkinsonian animal model constitutes the best-characterized neurotoxin paradigm for elucidating the biochemical and pathological alterations seen in PD, faithfully replicating many but not all behavioral, biochemical and pathological features of the disorder including the specific loss of dopamin(DA)ergic neurons in the substantia nigra pars compacta (SNc) (Przedborski and Vila, 2003; Przedborski et al., 2004; Duty and Jenner, 2011; Zeng et al., 2018; Kin et al., 2019). Clinically, DA replacement therapy using levodopa remains the gold standard for symptomatic relief in PD patients. However, due to the well-documented limitations of long-term DA replacement therapy and its widespread debilitating side-effects, neuroprotective strategies that slow or halt the disease progression are urgently needed (Simuni et al., 2009a; Simuni et al., 2009b; Sarkar et al., 2016; Fabbri et al., 2020). Mitochondrial dysfunction, oxidative stress, neuroinflammation and impaired protein degradation are implicated as potential pathological mechanisms of PD, but key downstream signaling targets contributing to nigral dopaminergic neuronal degeneration are not well established. Thus, the identification of potential pharmacological targets has been actively explored in PD.

Several protein kinases are being increasingly evaluated as therapeutic targets for human disease (Ventura and Nebreda, 2006; Balakrishnan and Gandhi, 2012; Quan et al., 2017). Considering that some recent breakthrough anticancer drugs are kinase inhibitors, exploration of kinases as potential therapeutic targets provides renewed optimism for the development of novel neurotherapeutic agents. Reports of Fyn as an oxidative stress-sensitive kinase and our previous reports of Fyn as a mediator of neuroinflammatory and neurodegenerative signaling events emphasize the need to systematically investigate this kinase during DAergic degeneration (Saminathan et al., 2011; Panicker et al., 2015; Panicker et al., 2019). As a key Src family kinase (SFK), Fyn is well expressed in nervous tissue and is primarily localized in the cytoplasmic leaflet of the plasma membrane (Resh, 1998; Saito et al., 2010; Matrone et al., 2020). Three isoforms have been described. One common isoform is expressed in B-lymphocytes and the brain (Resh, 1998; Saito et al., 2010). Specifically, in the nervous system, Fyn is required for oligodendrocyte differentiation, axonal guidance, learning, memory and the stabilization of neuromuscular junctions (Belkadi and LoPresti, 2008; Brackenbury et al., 2008; Saito et al., 2010; Saminathan et al., 2020; Matrone et al., 2020). Pathological roles attributable to Fyn reveal its activation by reactive oxygen species (ROS) (Abe and Berk, 1999) as well as other stress responses associated with several disease conditions. Fyn has a pathological signaling role during inflammatory bowel disorders (Ban et al., 2008; Saksena et al., 2008). Fyn is also upregulated during ROS-induced signaling in the cardiovascular system (Beheshti et al., 2019). The loss of central nervous system progenitor cells upon exposure to pro-oxidative insults underscores Fyn’s degenerative roles (Abe and Berk, 1999; Sanguinetti et al., 2003; Frossi et al., 2007; Li et al., 2007; Ban et al., 2008; Saksena et al., 2008). Oxidative stress is unquestionably an important determining factor in the DAergic neuronal degeneration underlying PD (Miller et al., 2009; Cenini et al., 2019; Trist et al., 2019).

Studies from our lab have shown that the pro-apoptotic Fyn-mediated signaling cascade contributes to oxidative stress-induced cell death in DAergic neurons (Kaul et al., 2005b; Saminathan et al., 2011) and that Fyn is also a key mediator of neuroinflammatory signaling events contributing to PD’s pathology (Panicker et al., 2015; Panicker et al., 2019). Previously, Dunah et al. (2004) reported that Fyn null-mice were resistant to 6-hydroxydopamine-induced striatal lesions, necessitating a systematic investigation and characterization of the mechanistic underpinnings of Fyn in PD. Several investigators have previously reported Fyn and Src phosphorylation sites on α-synuclein (Ellis et al., 2001; Nakamura et al., 2001; Takahashi et al., 2003), further suggesting Fyn’s roles in PD. Remarkably, a recent GWAS independently found the *FYN* gene to be associated with PD (Nalls et al., 2019). Fyn has docking sites on PKCδ and over-expression of several SFKs have been associated with tyrosine phosphorylation of PKCδ and an associated change in its kinase activity (Benes and Soltoff, 2001; Crosby and Poole, 2003). Recently, Fyn and PKCδ functionally interact in augmenting microglia-mediated neuroinflammation (Panicker et al., 2015; Panicker et al., 2019).

Herein, we used cellular-molecular approaches coupled with pharmacological and genetic interventions to characterize Fyn’s role in both *in vitro* and *in vivo* neurotoxicant-based models of PD, including N27 DAergic neuronal cells, primary mesencephalic neurons, and Fyn knockout (KO) mice. We report that 1) Fyn was highly expressed in nigral DAergic neurons; 2) suppression of Fyn by tyrosine kinase inhibitor or RNAi-mediated knockdown (KD) attenuated PKCδ Y311 phosphorylation and its proteolytic activation as well as DAergic neuronal apoptosis; and 3) Fyn KO mice were significantly protected from MPTP-induced DAergic degeneration and locomotor deficits, thereby providing overall credence to the premise that Fyn could serve as a novel pharmacological target of PD.

## Materials and Methods

### Chemicals

The p60^Src^ tyrosine-specific kinase inhibitor (TSKI) peptide was manufactured by the Iowa State University Protein Facility. The chemicals 3,3′-diaminobenzidine (DAB), 1-methyl-4-phenylpyridinium (MPP^+^), MPTP, and ATP were purchased from Sigma-Aldrich (St. Louis, MO). The primary antibodies used in this study were PKCδ, PKCδ-Y311-phospho-specific, Fyn (rabbit polyclonal, Santa Cruz Biotechnology, Santa Cruz, CA), tyrosine hydroxylase (TH) (both mouse monoclonal and rabbit polyclonal, Chemicon International, Temecula, CA), and β-actin (mouse monoclonal, Sigma). IRDye 800-conjugated anti-rabbit (Rockland Immunochemicals, Gilbertsville, PA) and Alexa Fluor 680-conjugated anti-mouse (LI-COR, Lincoln, NE) secondary antibodies were used. The caspase substrate Ac-DEVD-AFC was obtained from Bachem Biosciences (King of Prussia, PA) and {γ-^32^P}ATP was purchased from Perkin-Elmer Life Science (Boston, MA). The Bradford protein assay kit was purchased from Bio-Rad Laboratories (Hercules, CA). Neurobasal medium, B27 supplement, RPMI 1640 medium, fetal bovine serum, l-glutamine, penicillin and streptomycin, and Sytox green dye were purchased from Invitrogen (Carlsbad, CA). The Fyn kinase substrate was purchased from Enzo Life Sciences (Plymouth Meeting, PA).

### N27 Dopaminergic Neuronal Cell Culture

Immortalized rat mesencephalic N27 DAergic neuronal cells represent a homogenous TH-positive (TH^+^) cell line, which has been widely used as an *in vitro* model for PD (Kitazawa et al., 2004; Kaul et al., 2005b; Sun et al., 2005; Jin et al., 2011; Song et al., 2019). N27 cells were cultured in RPMI 1640 medium supplemented with 10% fetal bovine serum, 2 mM l-glutamine, 50 units of penicillin and 50 μg/ml of streptomycin. The cells were maintained in a humidified atmosphere with 5% CO_2_ at 37°C as described previously (Gordon et al., 2016a).

### Mouse Primary Mesencephalic Neuronal Cultures and Treatment

Primary mesencephalic neuronal cultures were prepared from the ventral mesencephalon of gestational 16- to 18-day-old (E16 to E18) mouse embryos from Fyn wild-type (+/+) and Fyn knockout (−/−), as described previously (Panicker et al., 2015) because the high number of TH^+^ neurons in the ventral mesencephalon, as well as, DA uptake, DA levels and striatal innervations are well established in E16 embryos. Mesencephalic tissues dissected from E16 to E18 mouse embryos were maintained in ice-cold Ca^2+^-free Hanks’ Balanced Salt Solution (HBSS) and then dissociated in HBSS containing trypsin with 0.25% EDTA for 30 min at 37°C. The dissociated cells were then plated at equal densities (0.7 × 10^6^ cells) on 12-mm coverslips pre-coated with 1 mg/ml poly-d-lysine. Cultures were grown in chemically defined conditions consisting of Neurobasal medium fortified with B27 supplements, 500 μM l-glutamine, 100 IU/ml penicillin and 100 μg/ml streptomycin. The cells were maintained in a humidified CO_2_ incubator (5% CO_2_ and 37°C). Half of the culture medium was replaced every 2 days. Approximately 6- to 7-day-old cultures were used for experiments. Briefly, primary mesencephalic neuronal cells were exposed to 5 μM MPP^+^ for 48 h and then processed for immunocytochemistry (ICC).

### Animal Studies

Heterozygous Fyn KO mice (Fyn (+/−) were a kind gift from Prof. Dorit Ron (University of California, San Francisco), and upon arrival, we established a successful breeding colony in our animal facility at Iowa State University. We generated Fyn (+/+) and Fyn (−/−) mice from breeding heterozygous Fyn (+/-) mice and then bred them as homozygous. Fyn (+/+) mice were used as wild-type controls (Fyn-WT). The Fyn KO mice (129-Fyntm1Sor/J, strain 129S7/SvEvBrd-Hprt<+>) are now available from Jackson Laboratory under stock number 002271. Use of animals and all animal-related procedures in this study (9–11–7235-M) were approved and supervised by the Institutional Animal Care and Use Committee at Iowa State University. All animals were housed under standard conditions for temperature (22 ± 1°C), humidity (relative, 30%), and a 12-h light/dark cycle. Mice were allowed *ad libitum* access to food and water.

#### MPTP Treatment

We used the acute MPTP treatment paradigm described in our previous publication (Ghosh et al., 2016a). Male Fyn (+/+) mice (8- to 12-week-old) were subjected to baseline behavioral activity and divided into 3 groups (32 animals/group). Sixteen animals from each group received four intraperitoneal injections of MPTP (18 mg/kg) at 2-h intervals and the remaining 16 animals received four intraperitoneal injections of 0.9% normal saline. Group 1 animals were euthanized at 3 h post-MPTP administration to extract the SN for biochemical assays including the Fyn kinase assay, as described below. Groups 2 and 3 animals were euthanized at 24 h and 7 days post-MPTP administration, respectively. Eight animals each from groups 2 and 3 were euthanized at 24 h and 7 days post-MPTP administration to extract the SN and striatum for neurobiochemical assays, as described below. The remaining eight animals from groups 2 and 3 were perfused and subjected to immunohistochemistry (IHC), as described below. Group 3 animals underwent behavioral studies on Days 5 and 6 post-MPTP administration. A similar study design was applied in parallel to 8- to 12-week-old Fyn (−/−) mice.

### Fyn Kinase Assay

Cells or ventral mesencephalon of gestational 16- to 18-day-old (E16 to E18) mouse embryos from Fyn wild-type (+/+) and Fyn knockout (−/−) were washed post-treatment with ice-cold PBS and resuspended in PKC lysis buffer as described in our previous publication (Kaul et al., 2005a). Crude protein (50 μg) was incubated with 150 μM Fyn kinase substrate (Biomol), 100 μCi of {γ-^32^P}a-ATP (Perkin Elmer)—Src-Mn-ATP cocktail, and Src reaction buffer (Upstate/Millipore, Billerica, MA) for 10 min at 30°C with agitation. To precipitate the Fyn kinase substrate peptide, 20 μL of 40% tricholoroacetic acid was added and 25 μL of the mixture was spotted onto a P81 phosphocellulose square. After spotting for 5 min, the squares were washed 5X in 0.75% phosphoric acid in PBS with a final wash step in acetone to fix the signals. The squares were transferred into a scintillation vial and the CPMA counts were read in a liquid scintillation system after adding 5 ml of scintillation cocktail to each vial.

### Western Blot

Cell lysates and brain lysates containing equal amounts of protein were separated on a 10–12% SDS-polyacrylamide gel and then transferred to a nitrocellulose membrane. The membranes were incubated with primary antibodies against TH (mouse monoclonal, 1:2000), Fyn kinase (rabbit polyclonal, 1:2000), PKCδ (rabbit polyclonal, 1:1,000), or *p*-Tyr-311-PKCδ (rabbit polyclonal, 1:1000). As described by Harischandra et al. (2015), the primary antibody treatment was followed by treatment with IRDye 800-conjugated anti-rabbit or Alexa Fluor 680-conjugated anti-mouse secondary antibodies (1:5,000) for 1 h at ambient temperature. Membranes were also developed with an HRP-conjugated secondary antibody followed by ECL detection as previously described (Anantharam et al., 2002). Western blot images were captured with the Odyssey IR imaging system (LI-COR, Lincoln, NE) or by using a scanner, and data were analyzed using either Odyssey 2.0 or ImageJ software.

### Enzymatic Assay for Caspase-3

A caspase-3 enzymatic assay was performed as described in our previous publications (Kaul et al., 2005b; Sun et al., 2007). After treatment, cells were washed in ice-cold PBS (pH 7.4) and resuspended in caspase lysis buffer at 37°C for 20 min. Lysates were centrifuged at 14,000 rpm. Cell-free supernatants were incubated with 50 μM Ac-DEVD-AMC (fluorometric caspase-3 substrate) at 37°C for 1 h. The formation of 7-amino-4-methylcoumarin (AFC), resulting from caspase-3 activity, was measured fluorometrically using a Gemini XS fluorescence microplate reader (Molecular Devices Inc.) at 400 nm excitation and 505 nm emission.

### siRNA Transfection

Transfections were performed as described in our previous publications (Jin et al., 2011; Song et al., 2019). Small interfering RNA (siRNA) targeted against the coding region of rat Fyn kinase and non-specific siRNA control were obtained from Ambion (Applied Biosystems, Austin, TX). N27 cells were transiently transfected with the specific and non-specific siRNA duplexes by the Amaxa Nucleofector kit (Lonza, Walkersville, MD). Using the kit, N27 cells were resuspended with transfection buffer to a final concentration of 4–5 × 10^6^ cells/100 μL and mixed with the siRNA duplexes. The final concentration of the siRNA was 5 nM. Electroporation was executed with an Amaxa Nucleofector instrument following the manufacturer’s protocol. The transfected cells were transferred to 6-well plates or T-175 flasks for 24–36 h before further treatments.

### Quantification of TH^+^ Cell Counts and Neuronal Processes

MetaMorph software, version 5.0 (Molecular Devices, Sunnyvale, CA), was used to count TH^+^ cells and neuronal processes in primary neurons from each coverslip. First, the images were thresholded and neurons were counted using the Integrated Morphometry Analysis (IMA) function in MetaMorph. The data were collated and analyzed in Excel (Microsoft, Richmond, WA). Neuronal process length was marked by applying the region and length measurement function in IMA. These data were also exported to and analyzed in Excel. TH^+^ cell counts and neuronal processes were measured in at least six individual cultures for each treatment. This is a modified version of a previous method used for quantification of neuronal processes (Yang et al., 2004; Zhang et al., 2007a; Gordon et al., 2016a).

### High-Affinity [^3^H] Dopamine Uptake Assay

As we described in Ghosh et al. (2013), cells in each well were washed twice with 1 ml of Krebs-Ringer buffer (16 mM NaH_2_PO_4_, 16 mM Na_2_HPO_4_, 119 mM NaCl, 4.7 mM KCl, 1.8 mM CaCl_2_, 1.2 mM MgSO_4_, 1.3 mM EDTA, and 5.6 mM glucose; pH 7.4). The cells were then incubated with 25 nM [^3^H] DA in Krebs-Ringer buffer (0.4 ml/well) for 20 min at 37°C. Non-specific uptake of DA was determined in parallel wells incubated with tritiated DA and 10 μM mazindol, an inhibitor of neuronal high-affinity DAergic uptake. Briefly, the cells were triple-washed with ice-cold Krebs-Ringer buffer (1 ml/well) and lysed with 1 N NaOH (0.5 ml/well). After washing, the lysates were mixed with 5 ml of scintillation fluid (ScintiVerse TM Cocktail, Fisher Scientific, Fair Lawn, NJ). Specific DAergic uptake was calculated by subtracting the amount of radioactivity observed in the presence of mazindol from that observed in the absence of mazindol, as measured with a liquid scintillation counter (Tri-Carb 4000; Packard, Meriden, CT).

### Locomotor Activity

Behavioral studies were performed using the computerized VersaMax animal activity monitoring system (model RXYZCM-16; AccuScan Instruments Inc., Columbus, OH) as described in our publications (Harischandra et al., 2019; Panicker et al., 2019). Each partitioned open-field arena was 20 × 20 cm, made of clear Plexiglas and covered with a Plexiglas lid with holes for ventilation. Infrared monitoring sensors were located every 2.54 cm along the perimeter (16 infrared beams along each side) and 2.5 cm above the floor. Two additional sets of 16 sensors were located 8.0 cm above the floor on opposite sides. Data were collected and analyzed by a VersaMax analyzer (model CDA-8, AccuScan Instruments Inc.), which in turn sent information to a computer where it was saved for future analyses. Locomotor activity was presented as horizontal, vertical, stereotypy movements and their associated parameters. All the data are expressed in raw values where the vehicle-treated group was used as the control group (mean ± S.E.M.; *n* = 6–8). The readings were obtained 5 days after MPTP or vehicle treatment in a 15-min session. Forced locomotor coordination was assessed on a Rota-Rod (AccuScan) with the rotating 3-cm diameter rod calibrated to a constant speed of 20 rpm. Time spent on the rod was measured for 20 min maximum during a maximum of five trials. Both instruments use infrared sensors to record beam breaks in both horizontal and vertical planes (VersaMax) or to record falls (Rota-Rod).

### Immunocytochemistry

ICC was performed as described in our previous publications (Gordon et al., 2016a; Harischandra et al., 2019; Sarkar et al., 2020).

#### Primary Mesencephalic Neurons

After treatment, neurons were first fixed with 4% paraformaldehyde (PFA) solution, and nonspecific sites were blocked with 2% bovine serum albumin, 0.1% Triton X-100 and 0.01% Tween-20 in phosphate-buffered saline (PBS) for 1 h. Cells were then incubated overnight at ambient temperature with the primary antibodies directed against TH (1:1,600 dilution) and Fyn kinase (1:500), followed by incubation with Alexa fluor 568 (red; 1:2,000) and Alexa fluor 488 (green; 1:2,000) secondary antibodies for 90 min at ambient temperature. Following secondary antibody treatments, cells were incubated at RT for 5 min with 10 μg/ml of the nuclear stain Hoechst 33342. Cultures from each batch of embryos were processed under identical conditions, including both primary and secondary antibody concentrations and incubation times. The coverslips containing the stained cells were washed with PBS, mounted on slides, and photomicrographed with a SPOT digital camera (Diagnostic Instruments, Inc, Sterling Heights, MI) attached to a Nikon Eclipse TE2000-U inverted fluorescence microscope (Tokyo; Japan).

#### Fyn and TH Immunofluorescence Staining of Nigral Neurons

Fixed brains from the 24-h study were cryosectioned and 5-μm sections from regions of interest were subjected to TH and Fyn immunostaining. After blocking non-specific sites, nigral sections were permeabilized with blocking buffer for 1 h at RT, then incubated overnight at RT with the primary antibodies directed against TH (1:1,600 dilution) and Fyn kinase (1:500), followed by incubation with Alexa fluor 568 (red; 1:2,000) and Alexa fluor 488 (green; 1:2,000) secondary antibodies for 90 min at RT. Following secondary antibody treatments, sections were incubated at RT for 5 min with 10 μg/ml of the nuclear stain Hoechst 33342. ProLong Gold antifade mounting medium (Molecular Probes) was used to mount sections on slides. Photomicrographs of sections were captured at 30X magnification using a SPOT digital camera coupled to an inverted fluorescence microscope (Nikon Eclipse TE2000-U).

### DAB Immunostaining and Stereological Analysis of Nigral Sections

Mouse brains were removed following perfusion with 4% PFA solution, post-fixed in 4% PFA solution and used for TH IHC and stereological analysis as described previously (Thiruchelvam et al., 2004; Zhang et al., 2007a; Harischandra et al., 2019). Fixed brains from the 7-day study were cryosectioned and 30-μm sections from regions of interest were collected in cryoprotectant. Sections were washed in PBS at RT before immunostaining and then incubated with an anti-TH antibody. Specifically, immunoperoxidase staining of striatal and SNc sections involved a biotinylated secondary antibody incubated with avidin peroxidase (ABC Vectastain Kit, Vector Laboratories, Burlingame, CA) and then DAB to stain the sections brown. The total number of TH^+^ neurons in the SNc was counted using an optical fractionator as described by Thiruchelvam et al. (2004). After delineating the region at low magnification (2X), every sixth section from the entire SN was sampled at higher magnification (100X objective) using Stereo Investigator software (MBF Bioscience, Williston, VT).

### HPLC Analysis of Neurotransmitters

Striatal levels of DA and its metabolites 3,4-dihydroxyphenylacetic acid (DOPAC) and homovanillic acid (HVA) were determined by HPLC with electrochemical detection (Ghosh et al., 2013). Briefly, neurotransmitter extracts obtained by centrifuging samples in an ice-cold antioxidant solution (0.1 M perchloric acid containing 0.05% Na_2_EDTA and 0.1% Na_2_S_2_O_5_) at 14,000 rpm in 0.22-μm-filtered spin tubes were loaded into a thermostatted autosampler (ESA model 542; Bedford, MA) together with solutions of catecholamine standards (Sigma Aldrich) that were diluted to a final working concentration of 50 pg/μL. The autosampler maintained samples at 4°C before injecting 20 μL of each sample at a flow rate of 0.7 ml/min. After the mobile phase (MD-TM, Thermo Fisher) first passed through a guard cell (ESA model 5020), DA, DOPAC and HVA were separated isocratically in a reversed-phase column coupled to a microdialysis cell (ESA model 5014A) and a Coulochem (ESA model 5100A) electrochemical detection system. The data acquisition and analysis were performed using EZStart HPLC Software (ESA Inc.). The DA, DOPAC and HVA levels were quantified as ng/mg of tissue weight.

### Data Analysis

Prism 4.0 software (GraphPad Prism, San Diego, CA) was used to compare treatment groups using either Student’s t-test or one-way ANOVA with Tukey’s post hoc test. Mean differences were deemed to be statistically significant when *p ≤* 0.05.

## Results

### PKCδ-Y311 Phosphorylation by Activated Fyn Kinase Mediates Apoptosis in N27 DAergic Neuronal Cells

**FIGURE 1:**
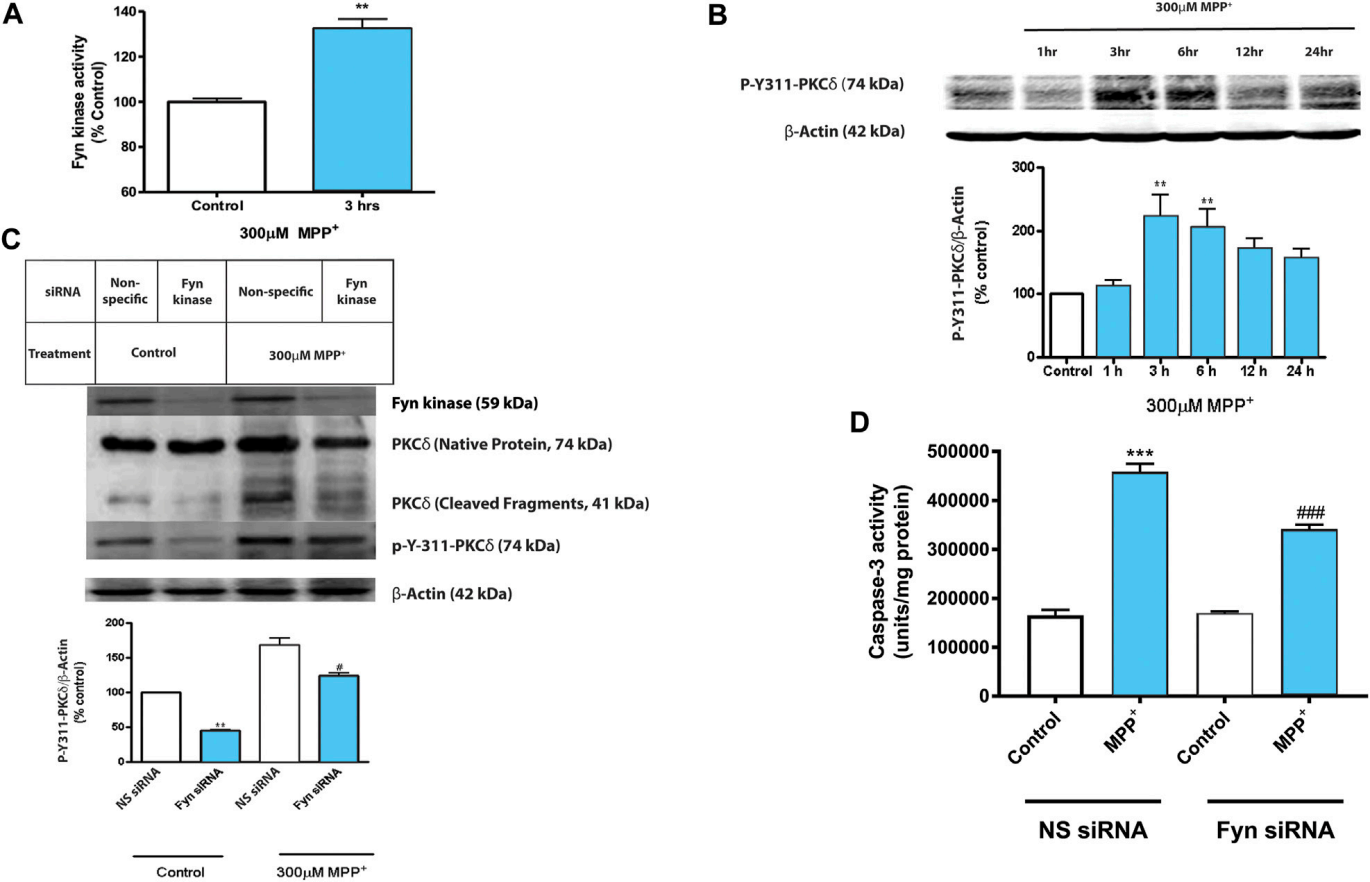
Activated Fyn kinase phosphorylates PKCδ Tyr 311 in N27 dopaminergic neuronal cells during MPP^+^ treatment**.** N27 cells were exposed to 300 μM MPP^+^ for up to 24 h. **(A)** Fyn kinase activity assay of cell lysates that were incubated with a Fyn kinase-specific substrate following 3 h treatment with MPP^+^. **(B)** Western blot and densitometric analysis showing time-dependent Y311 phosphorylation of PKCδ in MPP^+^-treated N27 cells. Representative Western blot images from 3 independent experiments are shown. **(C)** Western blot and densitometric analysis showing the effects of MPP^+^ at the 3-h time-point on protein expression levels of Fyn, PKCδ and p-Y311-PKCδ in N27 cells transiently transfected with Fyn-specific siRNA or non-specific (NS) siRNA. **(D)** Caspase-3 activation assay of the transfected cells described above at 24 h time-point. Data are represented as mean ± SEM from three independent experiments. Statistically significant treatment differences indicated by **p < 0.01, ***p < 0.001 when comparing control with MPP^+^; and ^#^ *p* ≤ 0.05, ^###^ *p* < 0.001 when comparing Fyn and non-specific siRNA for the MPP^+^ treatments, *n* = 3–4 per group.

We reported previously that the pro-oxidant hydrogen peroxide (H_2_O_2_) can induce Y311-PKCδ phosphorylation (Kaul et al., 2005b). Attenuation of PKCδ-Y311 phosphorylation with Fyn kinase inhibitors and overexpression of a dominant-negative PKCδ^Y311F^ mutant rescued N27 DAergic cells from H_2_O_2_-induced caspase-3-mediated PKCδ proteolytic activation and neuronal cell death (Kaul et al., 2005b). We had also reported that pharmacological and genetic ablation of Fyn activity rescued N27 DAergic neuronal cells from cell death induced by the organochloride pesticide dieldrin (Saminathan et al., 2011). In this study, we characterized the Fyn-PKCδ signaling cascade using highly relevant *in vitro* and *in vivo* models of PD. Treatment with MPP^+^, the toxic metabolite of the Parkinsonian toxicant MPTP, significantly increased the activity of Fyn in N27 DAergic cells at 3 h post-treatment (Figure 1A), which coincided with significantly increased phosphorylation of PKCδ-Y311 (Figure 1B). Since the activation time points of Fyn kinase and PKCδ Y311 phosphorylation coincided, we investigated the functional roles of Fyn activation and Y311-dependent proteolytic activation of PKCδ during MPP^+^-induced DAergic cell death. We transiently transfected N27 cells with non-specific (NS) or Fyn-specific siRNA, and then 24 h post-transfection, the cultures were exposed to MPP^+^ for 24 h. The siRNA knockdown (KD) of Fyn kinase attenuated MPP^+^-induced proteolytic activation of PKCδ as well as PKCδ-Y311 phosphorylation (Figure 1C). Since caspase-3 has been shown to mediate the proteolytic activation of PKCδ, we determined if Fyn-specific siRNA also significantly blocks MPP^+^-induced caspase-3 activation. The siRNA KD of Fyn did attenuate MPP^+^-induced caspase-3 activation (Figure 1D). Non-specific siRNAs did not have any effect on MPP^+^-induced proteolytic activation of PKCδ, PKCδ-Y311 phosphorylation or caspase-3 activation. Taken together, these results demonstrate that Fyn phosphorylates PKCδ on Y311 and this priming signaling event is necessary for caspase-3-mediated PKCδ proteolytic activation and downstream cell death signaling events.

### TSKI Protects Primary DAergic Neurons Against MPP^+^-Induced Degeneration

**FIGURE 2:**
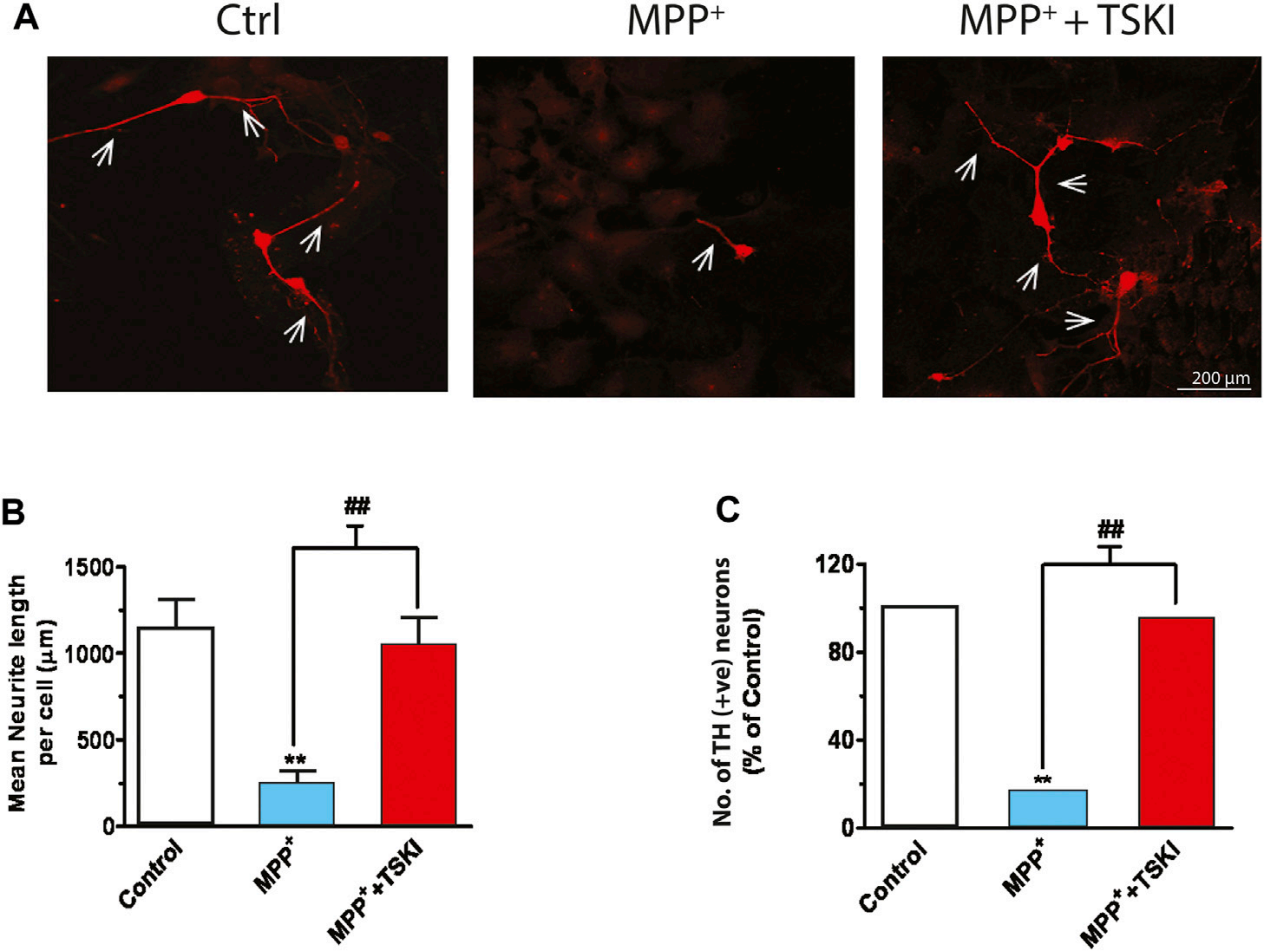
TSKI attenuates MPP^+^-induced dopaminergic neuronal degeneration in mesencephalic primary cultures. **(A)** Immunofluorescence images showing discernible attenuation of neurodegeneration in primary mesencephalic neurons exposed to 5 μM MPP^+^ for 48 h in the presence or absence of 5 μM TSKI. **(B)** MetaMorph image analysis of neuronal process lengths of the primary mesencephalic neurons described above. **(C)** MetaMorph image analysis of TH^+^ cell count. Statistically significant treatment differences indicated by **p < 0.01 for Control v. MPP^+^ and ^##^ *p <* 0.01 MPP^+^ v. MPP^+^ + TSKI (*n* = 4).

Our earlier findings demonstrated that pharmacological tyrosine kinase inhibitors—Genistein and the p60^Src^ TSKI peptide—attenuated oxidative stress-induced phosphorylation of PKCδ-Y311 and its proteolytic activation, suggesting a possible role for a Src family kinase located upstream in the PKCδ-mediated apoptotic-signaling cascade (Kaul et al., 2005b). Here, we examined if TSKI rescues TH^+^ neurons in primary mesencephalic cultures during MPP^+^ toxicity. After exposing primary mesencephalic cultures to 5 μM MPP^+^ in the presence or absence of 5 μM TSKI for 48 h, TH ICC of the primary neurons revealed that MPP^+^ treatment induced approximately a 90% loss of both TH^+^ cells and neurite processes (Figure 2A), indicating extensive DAergic degeneration. The average neurite length of TH^+^ neurons in the MPP^+^-TSKI-treated group was significantly longer than the processes of neurons treated with MPP^+^ alone (Figure 2B), and the TSKI treatment significantly protected against the MPP^+^-induced loss of TH^+^ neurons (Figure 2C). These data suggest that the pharmacological inhibitor TSKI is neuroprotective against MPP^+^-induced DAergic neurodegeneration in primary mesencephalic cell cultures.

### Fyn-Deficient Primary DAergic Neurons Are Resistant to MPP^+^-Induced Neurodegeneration

**FIGURE 3:**
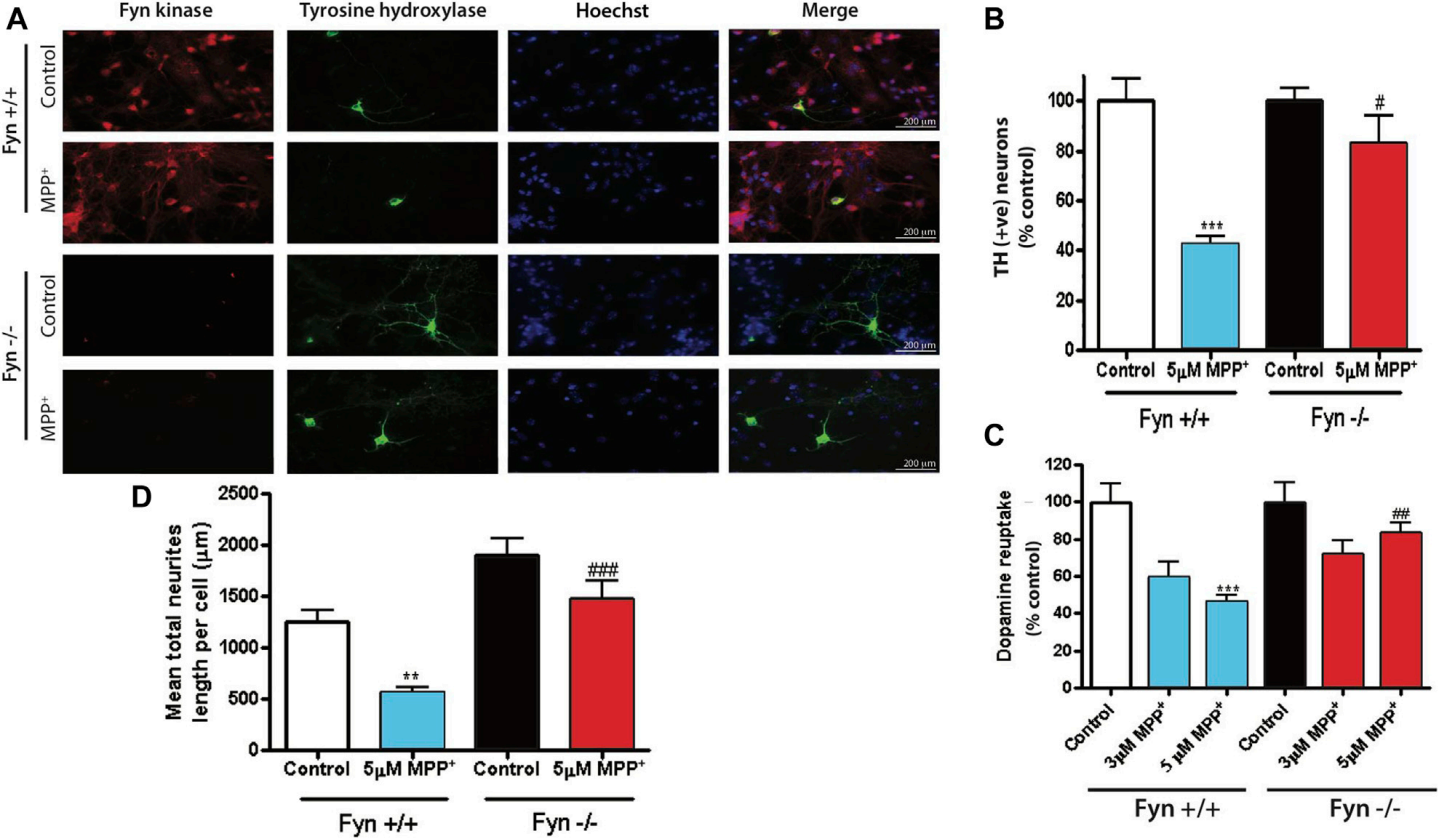
Fyn-deficient primary dopaminergic neurons are protected against MPP^+^-induced neurodegeneration. **(A)** Immunofluorescence images showing Fyn kinase (in red), tyrosine hydroxylase (in green) and nuclei (in blue) of primary mesencephalic neurons cultured from Fyn wild-type (WT) and knockout (KO) mice exposed to 3 or 5 μM MPP^+^ for 48 h. **(B, C)** MetaMorph image analysis of **(B)** TH^+^ cell count and **(C)** neuron process lengths in WT and Fyn KO primary mesencephalic cultures 48 h after exposure to 5 μM MPP^+^. ***D,*** High-affinity [^3^H] dopamine uptake by Fyn WT and KO midbrain primary neurons after exposure to 3 and 5 μM MPP^+^. Statistically significant treatment differences indicated by **p < 0.01, ***p < 0.001 when comparing control with MPP^+^; and ^#^ *p* ≤ 0.05, ^##^ *p* < 0.01, ^###^ *p* < 0.001 when comparing Fyn WT and KO for the MPP^+^ treatments, *n* = 4 per group.

To further substantiate the role of Fyn in PD-associated DAergic degeneration, we extended our investigation to primary mesencephalic DAergic neurons derived from wild-type (WT) and Fyn KO embryos. WT and Fyn-deficient DAergic neurons were exposed to 5 μM MPP^+^ for 48 h and the extent of DAergic degeneration was determined by TH ICC. As expected, the MPP^+^ treatment significantly reduced the number of TH^+^ WT neurons while TH^+^ Fyn KO neurons were significantly preserved (Figures 3A,B). Concomitantly, the mean lengths of TH^+^ neurites or neuronal processes in MPP^+^-treated Fyn KO neurons were significantly longer than those of MPP^+^-treated WT neurons (Figure 3C). We further performed [^3^H]-DA uptake assays since the functional capacity of DAergic neurons is well correlated with the DA reuptake capacity of their neurites (Liu et al., 2000). For this, we treated WT and Fyn KO primary neurons with 3 and 5 μM MPP^+^ for 48 h. As expected, MPP^+^ treatment significantly reduced DAergic uptake in cultures derived from the WT mice (Figure 3D), at both doses tested, whereas the cultures from Fyn KO mice were significantly resistant to any MPP^+^-induced deficit in [^3^H] DAergic reuptake. Taken together, these results clearly demonstrate that Fyn contributes to MPP^+^-neurodegenerative events in primary mesencephalic cell cultures.

### MPTP Activates Fyn Kinase and induces PKCδ-Y311 Phosphorylation in the Mouse SNc

**FIGURE 4:**
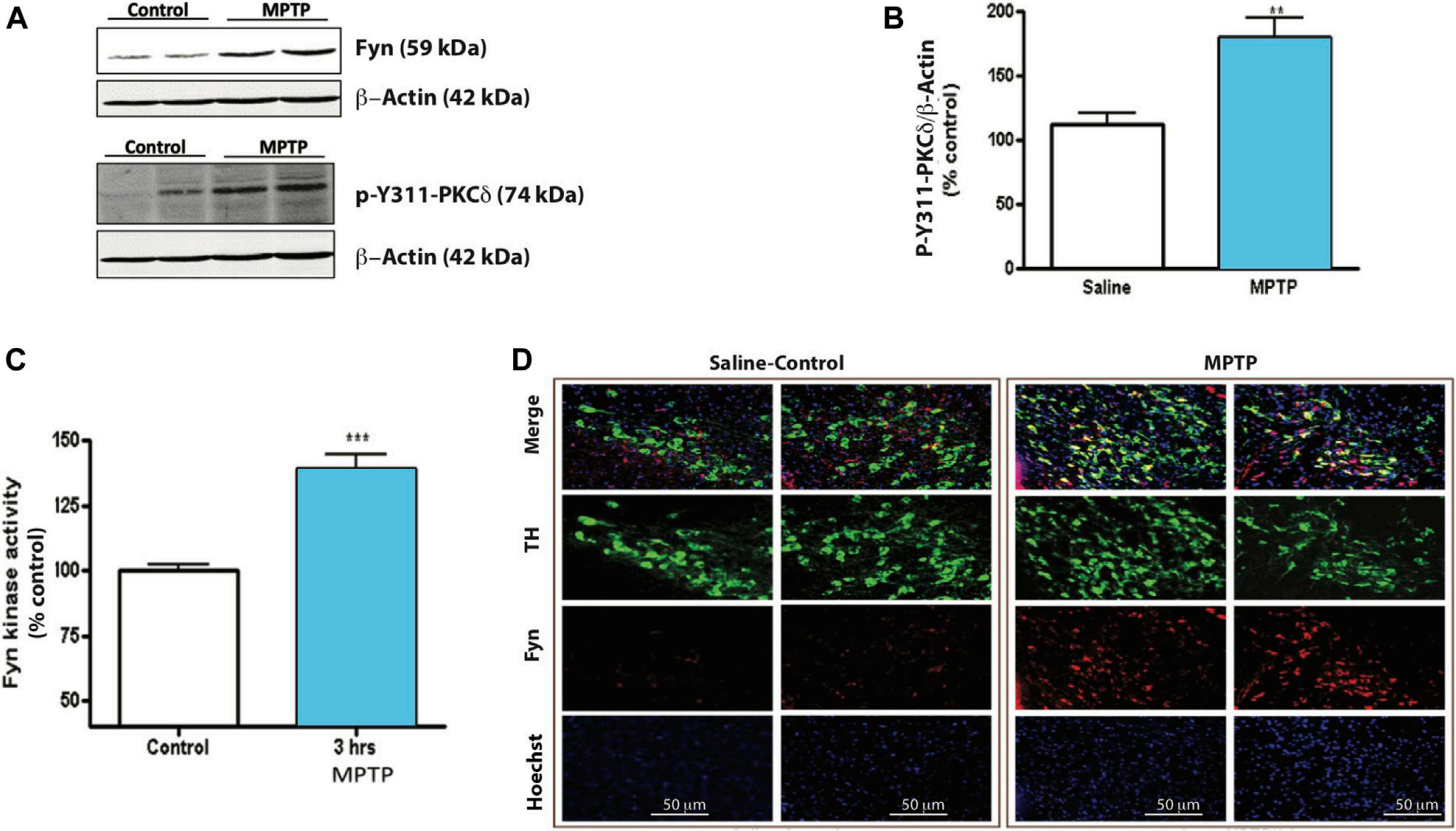
MPTP induces Fyn kinase activity and PKCδ Tyr 311 phosphorylation in mouse substantia nigra (SNc). **(A)** Western blot of SNc tissue lysates from mice 24 h after injection with vehicle or MPTP. Representative Western blot images from 3 independent experiments are shown. **(B)** Densitometric analysis of Western blot of p-Y311-PKCδ. **(C)** Fyn kinase activity assay of SNc tissue lysates incubated with a Fyn kinase-specific substrate. **(D)** Immunofluorescence images of TH-Fyn double immunostaining revealing that MPTP induces Fyn kinase expression in TH^+^ neurons. Data represent mean ± SEM from *n* = 3–6 mice per group. Statistically significant treatment differences indicated by **p < 0.01, ***p < 0.001; *n* = 4–6 mice per group.

Next, we used the acute MPTP treatment paradigm to elucidate *in vivo* Fyn kinase activation and PKCδ-Y311 phosphorylation 24 h following the acute treatment paradigm. Western blot analysis of SNpc lysates showed significant increases in both PKCδ-Y311 phosphorylation and Fyn protein levels (Figures 4A,B). Additionally, MPTP rapidly activated Fyn kinase at 3 h (Figure 4C), suggesting that in the *in vivo* system, MPTP-induced Fyn kinase activation precedes PKCδ-Y311 phosphorylation. IHC analysis of mouse SNc showed a co-localization of TH and Fyn expression within TH^+^ neurons and increased Fyn immunoreactivity after MPTP treatment (Figure 4D). Taken together, these results demonstrate Fyn expression in DAergic neurons and indicate that Fyn activation precedes PKCδ-Y311 phosphorylation in the SNc of MPTP-treated mice.

### Fyn-Deficient Mice Are Resistant to MPTP-Induced Locomotor Deficits

**FIGURE 5:**
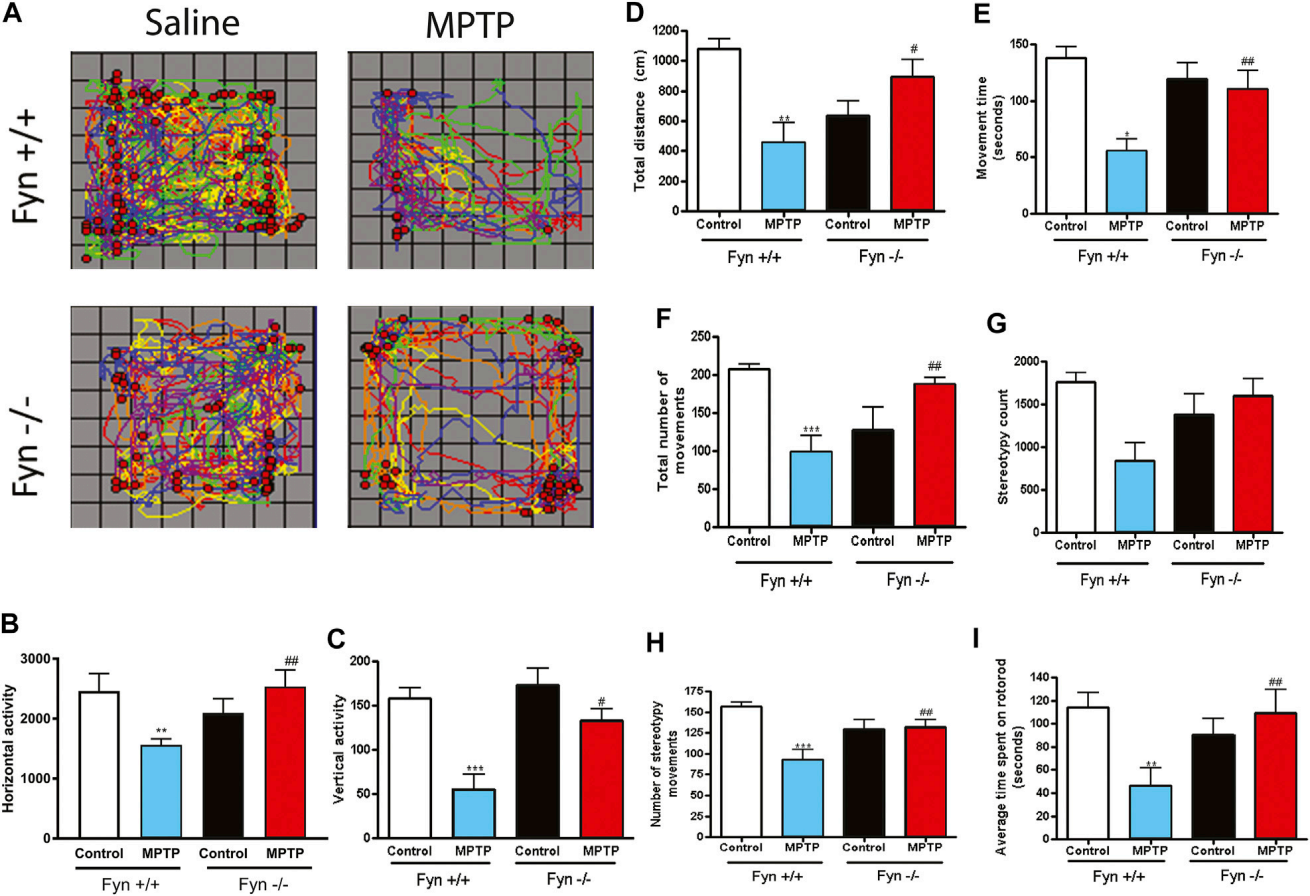
Fyn deficiency suppresses MPTP-induced locomotor deficits. Wild-type (WT) and Fyn knockout (KO) mice were injected with saline or MPTP and were then euthanized on day 7 after open-field and Rota-Rod performance was measured on days 5 and 6. **(A)** Track of horizontal (lines) and vertical (dots) movements. **(B)** Total horizontal and **(C)** vertical activities. **(D)** Total distance moved. **(E)** Movement time. **(F)** Total number of discrete horizontal movements. **(G)** Stereotypy count. **(H)** Number of stereotypy movements (bouts or episodes) and **(I)** Average time spent on Rota-Rod. Data are represented as mean ± SEM from 6 to 8 animals per group. Statistically significant treatment differences indicated by *p ≤ 0.05, **p < 0.01, ***p < 0.001 for WT control v. WT MPTP groups; and ^#^ *p* ≤ 0.05, ^##^ *p* < 0.01 for WT MPTP v. KO MPTP groups.

The measurement of spontaneous exploratory movements is a reliable indicator of locomotor deficits in animal models of PD. Therefore, prior to neurochemical analyses, saline- and MPTP-treated WT and Fyn KO mice were characterized for open-field and Rota-Rod performance. The cumulative horizontal and vertical locomotor activities are mapped in Figure 5A. Overall, the Fyn KO mice exhibited significantly milder MPTP-induced locomotor deficits. The signature behavior symptom of bradykinesia associated with PD is exemplified by the reduced signals of horizontal activity, vertical activity and movement numbers, as well as shorter latencies in falling from the Rota-Rod. Consistent with an extensive literature, MPTP induced significant decreases in most locomotor activities including horizontal activity (Figure 5B), vertical activity (Figure 5C), total distance (Figure 5D), movement time (Figure 5E), number of discrete horizontal movements (Figure 5F), count of stereotypic movements (Figure 5G), number of stereotypic episodes (Figure 5H) and average time spent on Rota-Rod (Figure 5I) in the WT mice. These findings further corroborate that the biochemical changes that afford neuroprotection in Fyn KO mice also translate to improvements in locomotor functional impairments in an acute MPTP model of PD.

### Fyn Deficiency Protects Striatal DA From MPTP-Induced Depletion

**FIGURE 6:**
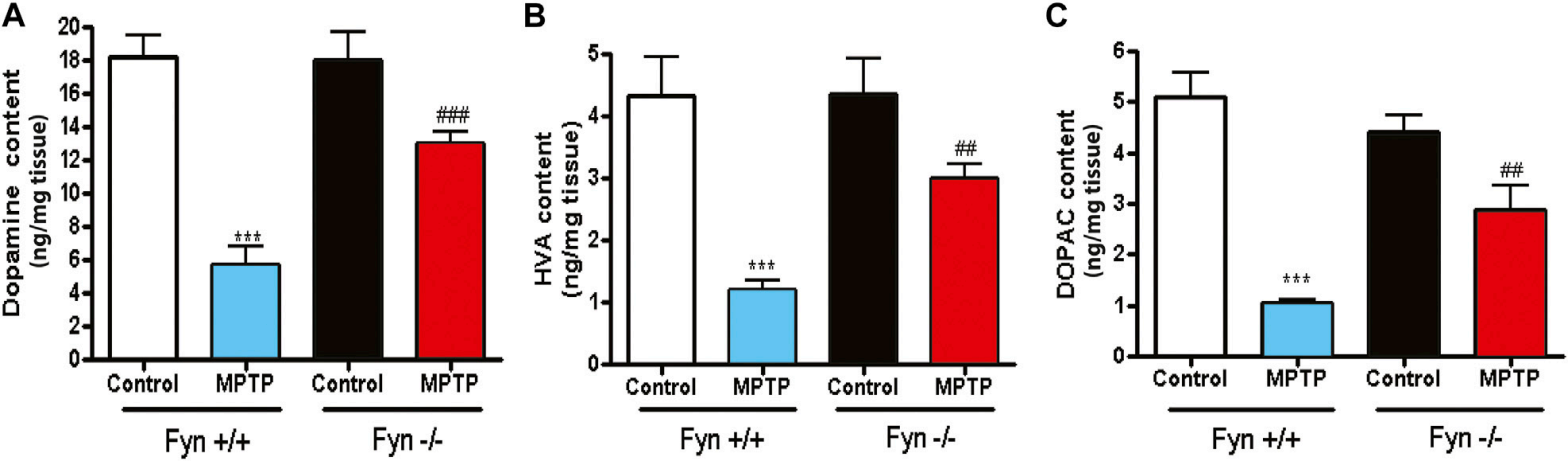
Fyn kinase deficiency attenuates MPTP-induced depletion of striatal dopamine (DA), 3,4-dihydroxyphenylacetic acid (DOPAC) and homovanillic acid (HVA). **(A)*–*(C)** Neurochemical analysis via HPLC of striatal tissue lysates from wild-type (WT) and Fyn knockout (KO) mice 7 days post saline or MPTP treatment. **(A)** DA. **(B)** HVA. **(C)** DOPAC. Data are represented as mean ± SEM from n = 10-12 mice per group. Statistically significant treatment differences indicated by ***p < 0.001 for WT control v. WT MPTP groups; and ^##^ *p* < 0.01, ^###^ *p* < 0.001 for WT MPTP vs. KO MPTP groups.

We next determined the neurochemical changes to striatal DA and its metabolites 7 days following MPTP injections to Fyn WT and Fyn KO mice. Fyn KO mice were significantly protected from MPTP-induced striatal DA depletion (Figure 6A). Similar results were observed with the DA metabolites HVA (Figure 6B) and DOPAC (Figure 6C). These results additionally reveal that Fyn KO mice are protected from DAergic neurotoxicity induced in an acute MPTP mouse model of PD.

### Fyn Kinase Phosphorylation of PKCδ Mediates DAergic Degenerative Signaling in Response to MPTP Treatment

**FIGURE 7:**
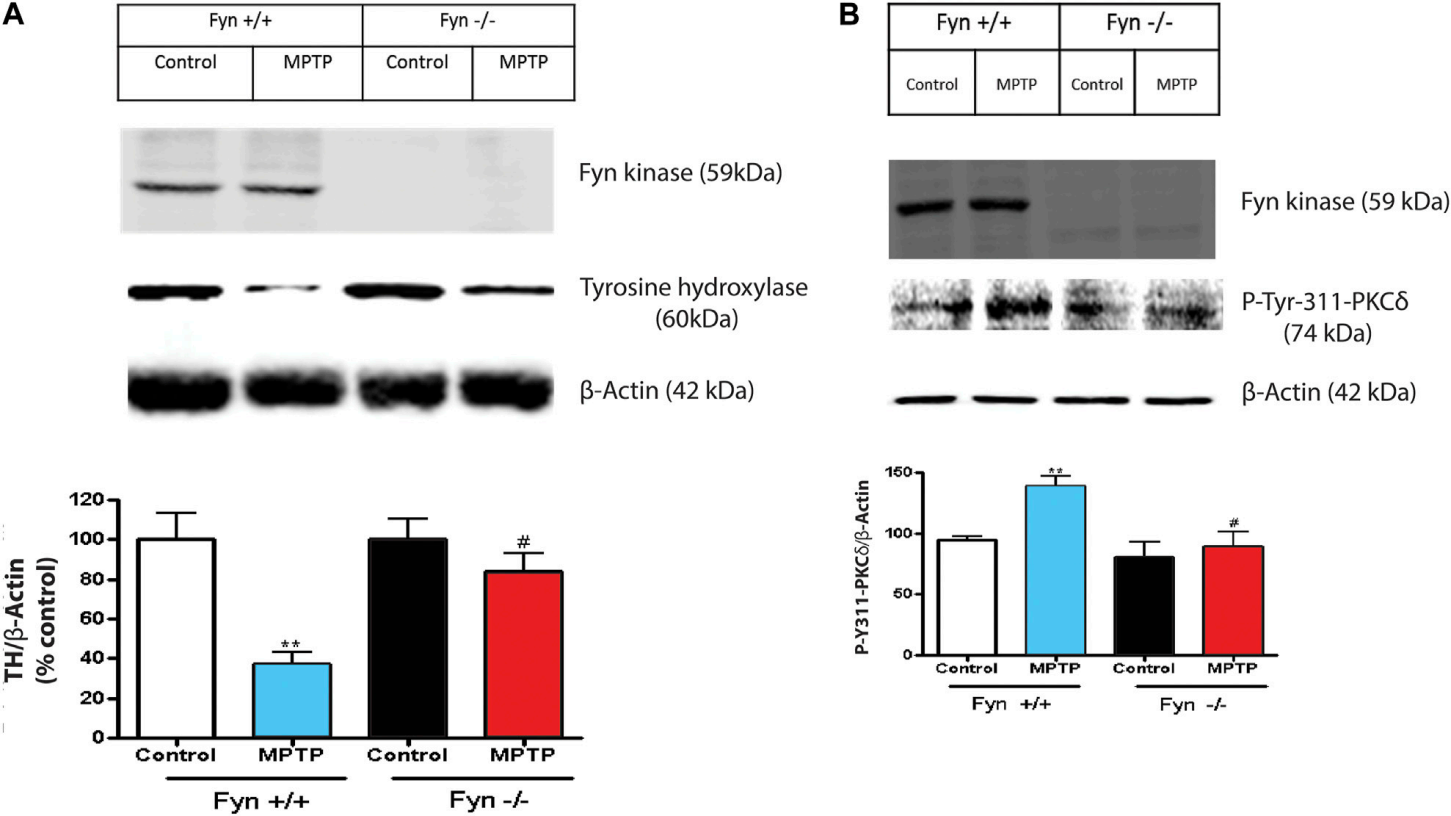
Fyn deficiency protects mice from MPTP-induced dopaminergic neurodegeneration. **(A)** Western blot of Fyn kinase and TH protein expression in SNpc lysates from wild-type (WT) and Fyn knockout (KO) mice post saline or MPTP treatment. Densitometric analysis of the immunoblots in 7A with relative densities of TH normalized to the densities of the corresponding β-Actin signals following 7 days post saline or MPTP treatment. Values (in arbitrary units) for TH are expressed as mean ± SEM for at least 6–8 mice per treatment. **(B)** Western blot of Fyn kinase and *p*-Tyr-311-PKCδ protein expression from the SNc following 24 h post saline or MPTP treatment**.** Densitometric analysis of the immunoblots normalized against β-actin. Statistically significant treatment differences indicated by **p < 0.01 for WT control v. WT MPTP groups; and ^#^ *p* ≤ 0.05 for WT MPTP v. KO MPTP groups.

Since Fyn phosphorylates PKCδ at site Y311 in dopaminergic neurotoxicity, (Figures 1A,B, and 4A,B), herein, we further examined the changes to PKCδ-Y311 phosphorylation following acute MPTP treatment in Fyn KO mice. WT and Fyn KO mice were euthanized at 24 h and 7 days following acute MPTP treatments. Fyn KO mice were significantly protected from MPTP-induced reductions in TH protein level after the 7-day treatment (Figure 7A), indicative of the protection of DAergic neurons. Immunoblotting for phosphorylation of PKCδ-Y311 from the SNpc lysates from MPTP-treated Fyn KO and WT mice revealed that Fyn-mediated PKCδ-Y311 phosphorylation was more evident in WT mice than in Fyn KO mice after 24 h treatment (Figure 7B). Collectively, these results provide further *in vivo* support that Fyn is involved in DAergic degeneration and demonstrate that Fyn-mediated PKCδ-Y311 phosphorylation activates the signaling axis leading to DAergic degeneration.

### Fyn Kinase Deficiency Protects Mice From Nigral DAergic Degeneration

**FIGURE 8:**
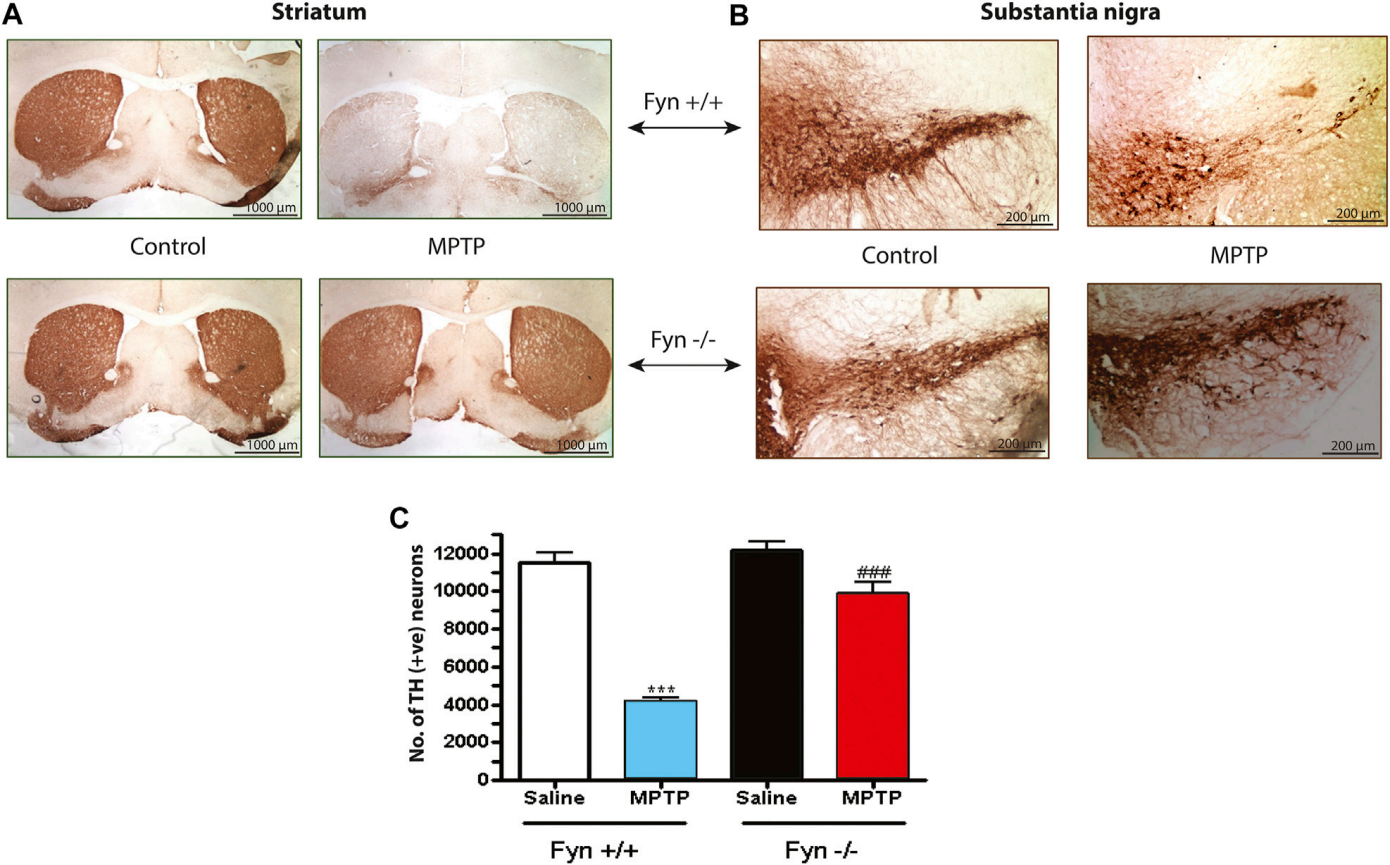
Fyn deficiency protects against the loss of substantia nigral (SNc) TH^+^ neurons and neurites in MPTP-treated mice. **(A)** Immunoperoxidase (DAB) staining of striatal TH sections from wild-type (WT) and Fyn knockout (KO) mice 7 days after the last MPTP injection. **(B)** TH DAB staining of SNc. **(C)** Total count of TH^+^ neurons in the SNc of groups exposed to either saline or MPTP, measured using unbiased stereology. Data are represented as mean ± SEM from *n* = 4 mice per group. Statistically significant treatment differences indicated by ***p < 0.001 for WT control v. WT MPTP groups; and ^###^ *p* < 0.001 for WT MPTP v. KO MPTP groups.

Because Fyn KO mice were effectively protected from the MPTP-induced loss of striatal DA and its metabolites, we also tested whether Fyn KO mice could be protected from MPTP-induced neurotoxicity in the SNc (Figures 8A,B). As expected, MPTP significantly reduced TH^+^ immunoreactivity in both the wild-type striatal terminals (Figure 8A) and SNc cell bodies (Figure 8B) and caused a 65–70% loss of SNpc TH^+^ neurons as revealed by stereological counting (Figure 8C). In contrast, TH^+^ immunoreactivity was well preserved in the striatum and SNc of MPTP-injected Fyn KO mice (Figures 8A,B). Likewise, the stereological count of TH^+^ neurons in MPTP-treated Fyn KO was significantly higher than that in the MPTP-treated WT mice (Figure 8C). Collectively, these data clearly suggest that Fyn mediates DAergic neurodegenerative processes in the MPTP model.

## Discussion

In the present study, we demonstrate that a key member of the Src family of kinases, Fyn, regulates the Y311 phosphorylation of PKCδ, which is a prerequisite for caspase-3-mediated proteolytic activation of PKCδ and downstream DAergic cell death signaling events. Following MPTP treatment and subsequent activation of Fyn, we demonstrate that suppressing Fyn activation by pharmacological inhibitor and siRNA KD can attenuate PKCδ-Y311 phosphorylation, PKCδ proteolytic activation and DAergic neuronal apoptosis. Additionally, Fyn KO mice were significantly protected from DAergic degeneration and locomotor deficits in the acute MPTP model of PD. Our studies reveal the key proapoptotic signaling cascade linking Fyn kinase to PKCδ signaling and that it is directly relevant to nigral DAergic degeneration.

Emerging roles of Fyn in neurodegenerative disorders demonstrate the necessity to understand the pathophysiological involvement of this important SFK. We previously reported that the substantia nigra has a high expression of Fyn and PKCδ in DAergic neurons as well as in activated microglial cells (Zhang et al., 2007b; Panicker et al., 2015; Gordon et al., 2016b; Panicker et al., 2019). Previous reports of Fyn’s multiple roles in the pathogenesis of Alzheimer’s disease suggest several unexplored aspects of this kinase during Parkinsonian neurodegeneration (Crews and Masliah; Haass and Mandelkow, 2010; Souter and Lee, 2010; Lee et al., 1998; Lee et al., 2004; Bhaskar et al., 2005; Derkinderen et al., 2005; Lee, 2005; Williamson et al., 2008; Hernandez et al., 2009). Compelling evidence of the neuroprotective abilities of SFK pharmacological inhibitors in *in vitro* models of PD also suggests the possibility of a pro-apoptotic role for this neuronal SFK (Kaul et al., 2005b). In addition to our previous report demonstrating that Fyn is a key player of DAergic degeneration by activating PKCδ via tyrosine phosphorylation (Saminathan et al., 2011), we also recently reported on Fyn’s role during microglial neuroinflammatory signaling events, thereby further broadening the significance of Fyn in PD (Panicker et al., 2015; Panicker et al., 2019). Several earlier reports from our lab show that the mitochondrial toxicants MPTP, dieldrin and MMT, oxidative stress, proteasomal dysfunction, divalent manganese, lipopolysaccharide, methamphetamine and vanadium induce mitochondrial dysfunction and the caspase-3-mediated proteolytic activation of PKCδ to induce DAergic degeneration (Song et al., 2010;Anantharam et al., 2002; Kanthasamy et al., 2003a; Kanthasamy et al., 2003b; Kitazawa et al., 2003; Anantharam et al., 2004; Yang et al., 2004; Kaul et al., 2005a; Kitazawa et al., 2005; Latchoumycandane et al., 2005; Kanthasamy et al., 2008; Afeseh Ngwa et al., 2009). Fyn and other SFKs have been shown to physically interact with PKCδ and to alter its kinase activity through its tyrosine phosphorylation, subcellular localization and substrate preferences (Gschwendt et al., 1994; Moussazadeh and Haimovich, 1998; Yamamoto et al., 2000; Blass et al., 2002; Joseloff et al., 2002; Kikkawa et al., 2002; Pula et al., 2005; Hall et al., 2007; Sumandea et al., 2008). In agreement with this, when we characterized the changes to Fyn kinase activation using *in vitro* and *in vivo* MPTP models of PD, we observed that Fyn activation precedes PKCδ-Y311 phosphorylation.

**FIGURE 9:**
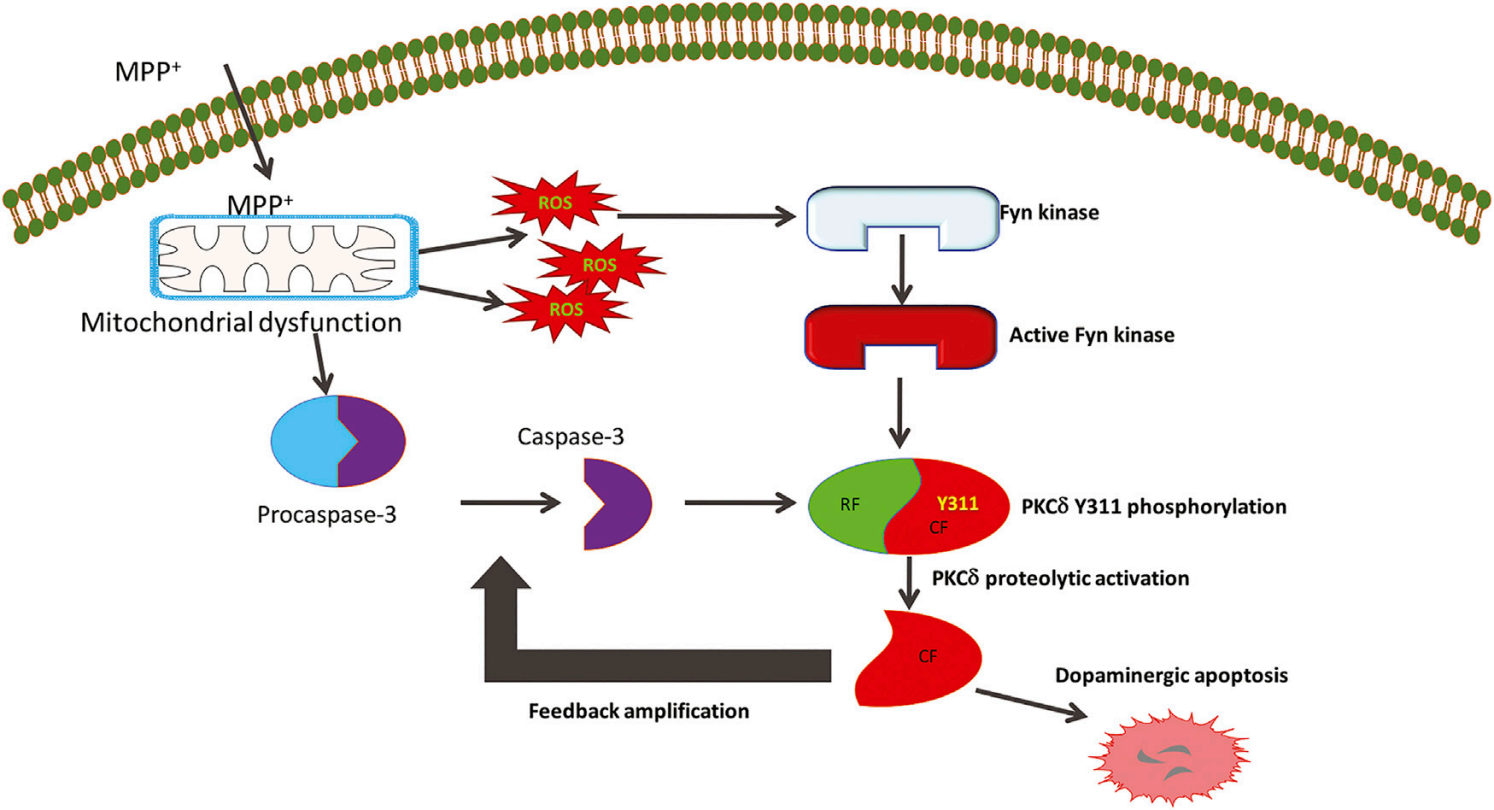
The role of Fyn kinase in mediating cell death signaling in Parkinson’s disease. The Parkinsonian toxicant MPTP’s active metabolite MPP^+^ produces mitochondrial dysfunction to generate reactive oxygen species (ROS) that trigger the activation of Fyn kinase. The early stress sensor molecule Fyn further phosphorylates PKCδ on the tyrosine 311 residue flanking the caspase-3 recognition site. This serves as the priming signaling event that allows caspase-3 to proteolytically activate PKCδ to initiate molecular events that cause dopaminergic cell death. The constitutively active catalytic fragment (CF) of PKCδ further amplifies the dopaminergic cell death events by feedback activation of caspase-3.

Molecular interactions between PKCδ with SFKs have been described in several disease models. SFKs have been shown to tyrosine-phosphorylate PKCδ at several tyrosine residues (Gschwendt et al., 1994; Blass et al., 2002; Rybin et al., 2007; Sumandea et al., 2008). Quite notably, Y311 and Y332 that flank the caspase-3 recognition site are phosphorylated upon exposure to the oxidative stress inducers H_2_O_2_ and ceramide, accompanied by increases in PKCδ kinase activity and its proteolytic activation (Konishi et al., 1997; Konishi et al., 2001; Okhrimenko et al., 2005; Balasubramanian et al., 2006; Murugappan et al., 2009). Phosphorylation of Y311 on PKCδ was associated with DAergic neuronal apoptosis, whereas in keratinocytes, phosphorylation of this tyrosine residue led to keratinocyte differentiation, suggesting alternative functional roles for PKCδ upon tyrosine phosphorylation, which is highly stimulus- and tissue-specific (Kaul et al., 2005b; Balasubramanian et al., 2006). By characterizing the changes to Fyn expression and activity in MPTP-induced PD-related degeneration, we show here that Fyn is highly expressed in the mouse SNc DAergic neurons and that genetic intervention against Fyn activity attenuated PKCδ-Y311 phosphorylation and DAergic apoptosis. Pharmacological intervention achieved similar results as well. Knowing that TSKI, an effective peptide inhibitor of SFKs, can completely ameliorate H_2_O_2_-, dieldrin- and MPP^+^-induced PKCδ-Y311 phosphorylation and proteolytic activation in N27 DAergic cells (Kaul et al., 2005b; Saminathan et al., 2011), we further show that TSKI protects TH^+^ neurons from MPP^+^-induced neurodegeneration in primary mouse mesencephalic neuronal cultures. Taken together, these observations suggest that Fyn lies upstream of PKCδ and contributes to PD pathogenesis by activating an apoptotic signaling cascade in DAergic neurons through PKCδ Y311 phosphorylation (Figure 9). Activation of Fyn followed by PKCδ-Y311 phosphorylation during MPP^+^ treatment, accompanied by TSKI’s neuroprotection against DAergic degeneration led us to further characterize Fyn-PKCδ signaling events in Fyn KO mice using an acute MPTP model of PD. It is noteworthy that Fyn-mediated PKCδ-Y311 phosphorylation might be only one of the several putative signaling pathways that may mediate apoptosis, which has been evident from the other research findings from our group spanning Fyn kinase (Panicker et al., 2015; Panicker et al., 2019).

In agreement with the neuroprotective abilities of SFK inhibition in primary neurons, we observed that primary DAergic neurons derived from Fyn KO mice are protected from MPP^+^-induced TH^+^ neurite shortening and neuron loss. The health of DAergic neurons is also exemplified by their ability to reuptake DA from the synapse (Liu et al., 2000; Bilsland et al., 2002; Zhang et al., 2007a). We additionally demonstrate that the primary DAergic neurons from Fyn KO mice still retain DA reuptake capacity following MPP^+^ treatment, suggesting that DA transporter proteins also may be functionally intact in the Fyn KO mice. Interestingly, we also observed that total neurite length was significantly increased in primary DAergic neurons from Fyn KO mice (Figure 3D), which is in agreement with Huang et al. (2017), who demonstrated that Fyn inhibition increased the length of the leading process ofmigrating cortical neurons. The most significant biochemical change in the MPTP-induced denervation of the striatum is the dramatic reduction in striatal DA. This upheaval is manifested as a characteristic motor dysfunction that leads to bradykinesia in the VersaMax open-field activity monitor and shortened latency to fall (deteriorated balance) on the Rota-Rod. Motor deficits in both of these tasks stem from the loss of striatal TH terminals and the consequent reduction of DA levels in the MPTP-treated striatum (Zhang et al., 2007a; Ghosh et al., 2007; Ghosh et al., 2009). Fyn KO mice were significantly protected from MPTP-induced motor deficits and accompanying this behavioral recovery was a significant increase in DA and its metabolite levels in the Fyn KO striatum (Figure 6). These findings collectively demonstrate the resistance of Fyn KO mice to MPTP toxicity.

It is noteworthy that targeting kinases as a pharmacological intervention is one of the most challenging aspects of clinical translatability. Particularly, because both Fyn kinase and PKCδ are expressed ubiquitously and have key biolological functions, targeting them is likely to generate adverse effects due to alterations to their house-keeping functions or biochemical signaling in vital tissues and organs. Moreover, invention of a highly Fyn-selective inhibitor is particularly challenging as Fyn shares several homologous domains with other members of Src family kinases. Nevertheless, we have recently demonstrated the utility of a small molecule inhibitor, dasatinib, in inhibiting Fyn kinase and rescuing mice from LPS-induced septic shock events (Saminathan et al., 2020). Moreover, saracatinib, a dual inhibitor of Src family kinases and Bcr-Abl tyrosine kinase, is now being pursued as an investigational molecule for Alzheimer’s pathology, and has shown immense therapeutic potential in preclinical models of Alzheimer’s disease (Nygaard et al., 2015; Nygaard, 2018).

In conclusion, our *in vitro* data and previous report (Saminathan et al., 2011) demonstrate that Fyn phosphorylation of Y311 on PKCδ activates the apoptotic signaling cascade in DAergic neurons in response to neurotoxic insults. As the next logical step, we utilized a Fyn KO transgenic mouse model of neurotoxicant-induced PD to confirm that the Fyn-PKCδ signaling axis mediates DAergic degeneration *in vivo*. Our finding that knocking out Fyn preserves nigral DAergic neurons and striatal dendrites against an acute neurotoxic insult supports the premise that Fyn kinase activation occurs early in the apoptotic process. Our previous reports of the dual roles of PKCδ as a mediator and amplifier of DAergic apoptosis and identification of Fyn in regulating neuroinflammatory events, as well as PKCδ activation by post-translational modification-dependent proteolytic activation are key findings in the understanding of Fyn’s role in DAergic neurodegeneration as depicted in Figure 9. Collectively, we demonstrate that the Fyn-PKCδ signaling axis plays a major pathological role in mediating nigral dopaminergic neurodegeneration and that the upstream regulator Fyn could serve as a potential therapeutic target for the development of disease-modifying therapies for the treatment of PD and other related neurodegenerative disorders.

## Data Availability

The raw data supporting the conclusions of this article will be made available by the authors, without undue reservation.
